# Dynamics of CD4+ and CD8+ Lymphocytic Inflammatory Infiltrates in Lupus Nephritis

**DOI:** 10.3390/ijms251910775

**Published:** 2024-10-07

**Authors:** Tudor Azoicăi, Elena-Roxana Avădănei, Simona-Eliza Giusca, Mihai Onofriescu, Adrian C. Covic, Cristina Gena Dascalu, Irina-Draga Căruntu

**Affiliations:** 1 Service de Rheumatology, CHU Helora, Avenue B. de Constantinople no. 5, 7000 Mons, Belgium; tudor.azoicai@helora.be; 2 Department of Morpho-Functional Sciences I—Histology, Pathology, “Grigore T. Popa” University of Medicine and Pharmacy, 16 University Street, 700115 Iași, Romania; simonaelizagiusca@gmail.com (S.-E.G.); irina.caruntu@umfiasi.ro (I.-D.C.); 3 Department of Pathology, “Dr. C. I. Parhon” Clinical Hospital, 50 Carol I Boulevard, 700503 Iași, Romania; 4 Department of Internal Medicine—Nephrology, “Grigore T. Popa” University of Medicine and Pharmacy, 16 University Street, 700115 Iași, Romania; mihai.onofriescu@umfiasi.ro (M.O.); accovic@gmail.com (A.C.C.); 5 Department of Nephrology, Dialysis and Renal Transplant Center, “Dr. C. I. Parhon” Clinical Hospital, 50 Carol I Boulevard, 700503 Iași, Romania; 6 Romanian Medical Science Academy, 1 I.C. Bratianu Boulevard, 030171 Bucharest, Romania; 7 Department of Preventive Medicine and Interdisciplinarity—Medical Informatics and Biostatistics, “Grigore T. Popa” University of Medicine and Pharmacy, 16 University Street, 700115 Iași, Romania; cristina.dascalu@umfiasi.ro

**Keywords:** lupus nephritis, CD4+ lymphocytes, CD8+ lymphocytes, systemic lupus erythematosus, SLE pathogenesis

## Abstract

Lupus nephritis (LN) is a common clinical manifestation of systemic lupus erythematosus (SLE). Our study aims to quantitatively analyze CD4+ and CD8+ lymphocytes in different areas and LN classes and describe a specific distribution pattern that is correlated with the severity of LN-specific lesions. In total, 53 LN renal biopsies were immunohistochemically investigated using anti-CD4 and anti-CD8 antibodies. T lymphocytes were counted in 3 areas, including intraglomerular, periglomerular, and interstitial regions. The severity of glomerular and tubulo-interstitial lesions was assessed using an original semi-quantitative algorithm based on the renal corpuscle score (RC_S) and the tubulo-interstitial score (TI_S). The number of CD8+ T lymphocytes was higher than that of CD4+ T lymphocytes in each of the three areas and in each LN class, showing statistically significant differences. ANOVA analysis of all LN classes showed significant differences between periglomerular and interstitial CD4+ and CD8+ T lymphocytes, respectively. Irrespective of location, the number of CD8+ T lymphocytes statistically correlates with the RC_S and the TI_S; no significant correlations were found between the number of CD4+ T lymphocytes and the RC_S and the TI_S for all three considered areas. Our data provide strong evidence supporting the major role of CD8+ lymphocytes in LN lesion progression, with CD4+ lymphocytes playing a limited role.

## 1. Introduction

Lupus nephritis (LN) as the most common severe manifestation of the systemic lupus erythematosus (SLE) is a significant contributor to SLE mortality [1,2,3].

Although in many cases the SLE diagnosis is based on clinical and paraclinical criteria, renal damage may be the first clinical onset in 15–20% of SLE patients [4,5]. In these cases, a renal biopsy is required to obtain an accurate diagnosis of the LN class and assess the activity and chronicity status, thus aiding in determining the prognosis and establishing a plan for therapeutic management. Also, any new evidence of disease progression (such as proteinuria, hematuria, abnormal urine sediment or increased serum creatinine) in a patient with SLE requires a new renal biopsy [6,7].

LN is a complex entity involving multiple pathogenic mechanisms [8,9] that act systemically through immune system changes and locally through kidney damage. Immunologically, LN is considered the prototype of autoimmune chronic pathology due to immune complex deposition and subsequent complement activation through both the classical and alternative pathways [10,11].

The autoimmune component of the pathogenesis includes aberrant B-lymphocyte activity, leading to autoreactivity [12], and the formation of abnormally activated T-lymphocytes [13]. This results in the production of autoantibodies, immune complexes, and cytokines, causing the development of an inflammatory response [11,14]. The repetitive sequences of the inflammatory response implicitly determine the chronic course of the disease. As a result, most patients with LN develop chronic kidney disease [15].

Studies in murine models have revealed abnormal signaling mechanisms and failure of immunological tolerance of B lymphocytes, and the AKT/mTOR signaling pathway plays a key role [16,17]. Research on signaling molecules in B lymphocytes has identified two inhibitory receptors, Lyn and CD22, which are essential in regulating their anomalous phenotype in LN [18]. Another inhibitory receptor, FCgammaRIIB, plays a critical role in preventing the development of this abnormal phenotype [18]. The deficiency of innate immunity and the failure of immune tolerance have led to the production of LN-associated self-antigens represented by nucleoproteins released during apoptosis, which activate Toll-like receptor 7 (TLR7) and 9 (TLR9) with a dual role in controlling B-lymphocyte behavior [19].

T lymphocytes are the most common inflammatory cells identified in renal lesions in both LN patients and mouse models of LN [20,21]. CD4+ T helper lymphocytes facilitate the production of autoantibodies. The individual balance between Th1 and Th2 subsets is responsible for different histological lesions [22], and the Th17 subset promotes inflammation [23,24,25]. Qualitative or quantitative defects in CD4+ T regulatory lymphocytes lead to dysfunctional immune responses [8,25]. On the other hand, CD8+ cytotoxic T lymphocytes act to eliminate autoreactive B lymphocytes. The decrease in autoreactive B lymphocytes attenuates or blocks the autoimmune sequence that leads to LN development, with the induction of peripheral tolerance also contributing to this process. Thus, by reducing the number of B lymphocytes, CD8+ cytotoxic T lymphocytes have an indirect role in peripheral tolerance [8].

Nonetheless, the involvement of double-negative T cells in LN is a hot topic; however, solid evidence for their role in LN pathogenesis is lacking [26]. Of note, there seem to be important differences in the functionality of T lymphocytes in human and experimental studies [25]. Several aberrant signaling pathways of T lymphocytes have been described in LN, including CD3ζ downregulation, PI3K-Akt-mTORC1 activation, and upregulation of calcium/calmodulin kinase IV (CaMKIV), Rho-associated protein kinase (ROCK), and protein phosphatase 2A (PP2A) [24]. The inappropriate regulation of these pathways has determined abnormalities in T cell differentiation, with increased release of IL-17, a proinflammatory cytokine, and a decreased production of IL-2, which is responsible for the proliferation and activation of these lymphocytes [8,9,24].

The presence of a tubulo-interstitial lymphocytic infiltrate is a characteristic feature of LN. However, only a limited number of morphological studies aimed to perform a quantitative characterization of lymphocyte populations and provided an analysis of their relationship to the severity of renal damage. As shown in published reports, the lymphocytic inflammatory infiltrate consists mainly of T lymphocytes (CD4+, CD8+, CD28+, and naïve cells), while B lymphocytes and macrophages are the minority component [27,28,29,30,31,32,33]. In the renal interstitium, B and T lymphocytes are disposed periglomerularly and peritubularly and organized in structures like those found in peripheral lymphoid organs [27,28,29,30,31,32,33].

In this context, the aim of our study was to quantitatively analyze the CD4+ and CD8+ lymphocyte populations in three different areas (periglomerular, intraglomerular, and interstitial areas) in different LN classes and to describe a specific distribution pattern that could be correlated with the severity of LN-specific lesions.

## 2. Results

### 2.1. Clinico-Pathological Characteristics of the Study Group

The study group comprised 53 patients, 44 females and 9 males, with an average age of 35 ± 13 years. The main clinico-pathological information at the time of the renal biopsy is shown in Table 1. In 48 out of 53 patients, the LN diagnosis was based on the clinico-biological profile. For these patients, the renal biopsy was performed to allow the histopathological assessment of renal lesions and the LN classification. The interval between the clinico-biological diagnosis of LN and renal biopsy was up to one year in 15 cases, between 1 and 3 years in 11 cases, between 4 and 5 years in 8 cases, and more than 5 years in 14 cases. In 5 patients, the diagnosis of LN established by clinico-biological criteria was confirmed from the outset by the renal biopsy.

According to the ISN/RSP classification, the analyzed cases were grouped as follows: mesangial proliferative LN class II, 2 cases (3.77%); focal LN class III, 4 cases (7.54%); diffuse LN class IV, 19 cases (35.84%); membranous LN class V, 22 cases (41.50%); and advanced sclerotic LN class VI, 6 cases (11.32%) (Table 1). Considering the small number of cases of classes II and III, we decided to report class II with class III.

In the study group, the therapeutic strategy consisted of glucocorticoid treatment alone in 14 (26.41%) patients and various immunosuppressive combinations (cyclophosphamide, cyclosporine, plaquenil, and mycophenolate mofetil) in the other 39 (73.58%) patients. 

At present, of the total study group followed from the time of LN diagnosis by renal biopsy, the outcome analysis showed that 28 (52.83%) patients had complete remission, 16 (30.18%) patients had partial remission, and 6 (11.32%) patients developed ESRD requiring dialysis; 3 (5.66%) patients died.

### 2.2. Comparative Analysis of the Clinico-Biological Profiles among the LN Classes

The comparative analysis (ANOVA test) indicated statistically significant differences in serum creatinine levels, eGFR (CKD-EPI), serum C3 complement levels, and triglyceride levels among the LN classes (Table 2). For serum creatinine level and eGFR (CKD-EPI), these results were sustained by significant differences between classes II + III, class IV and class V, separately, compared with class VI. On the other hand, for triglyceride and serum C3 complement levels, statistically significant differences were found in the comparison between class II + III and class IV and class V, separately; class IV and class VI; and class V and class VI (Table 2). No statistically significant differences between the other paraclinical data and LN classes were noticed (Table 2).

### 2.3. Specific Morphological Lesion Assessment Using Original Semi-Quantitative Scores in Lupus Nephritis Classes

Morphological characterization of the lesions, performed using semi-quantitative assessment of the lesions based on the corpuscular score (RC_S) and tubulo-interstitial score (TI_S), indicated significant morphological variability among the LN classes (Table 3). As for the RC_S, our data showed an upward trend in mean scores from classes II + III to classes IV and V (which had close mean scores) and then VI. For the TI_S, we found a similar pattern of the mean scores for the RC_S. 

### 2.4. Relationship among Semi-Quantitative Scores, rSLEDAI Score, and Lupus Nephritis Classes

The comparative analysis (ANOVA test) showed significant differences for both semi-quantitative scores when we compared classes II + III with class IV, class V, and class VI; class IV with class VI; and class V with class VI (Table 3). Additionally, for the renal SLEDAI score, we found statistically significant differences in the comparison between classes II + III and class V and class VI, between class IV and class V, and between class IV and class VI (Table 3).

### 2.5. Correlation between rSLEDAI Score and Semi-Quantitative Scores in Lupus Nephritis Classes 

The statistical study of correlation (Pearson correlation coefficients) revealed a statistically significant, positive, and moderate correlation between the RC_S and the rSLEDAI score at the level of the whole group (r = 0.361; *p* = 0.008). In contrast, there was a positive but weak and not statistically significant correlation between the TI_S and the rSLEDAI score at the level of the whole group (r = 0.239; *p* = 0.085) (Table 4).

### 2.6. CD4+ T Lymphocytes in Lupus Nephrites Classes

#### 2.6.1. Qualitative Evaluation

**Figure 1:**
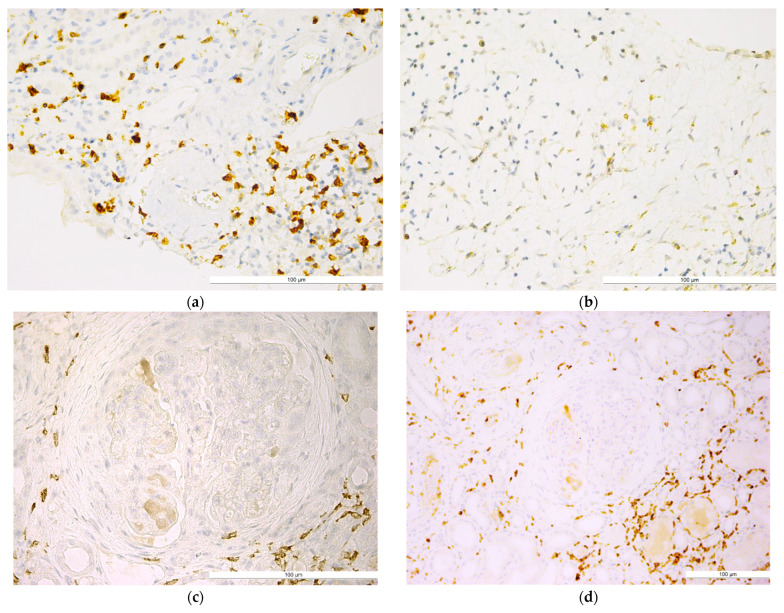

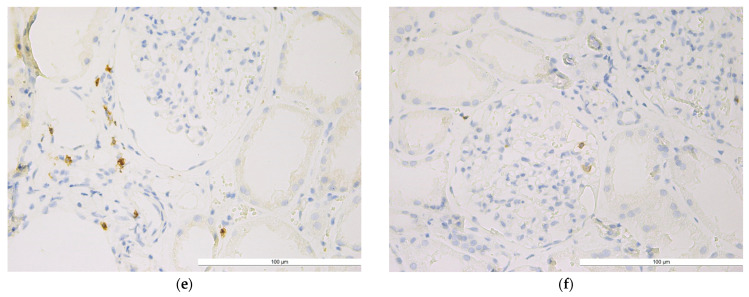
Lupus nephritis with (**a**) well-represented periglomerular and interstitial CD4+ T lymphocytes (IHC, anti-CD4, ×40); (**b)** CD4+ T lymphocytes in the renal interstitial space in an area of tubular atrophy (IHC, anti-CD4, ×40); (**c**) CD4+ T lymphocytes with concentric periglomerular localization (IHC, anti-CD4, ×40); (**d**) CD4+ T lymphocyte population in the periglomerular and interstitial areas (IHC, anti-CD4, ×20); (**e**) poorly represented periglomerular and interstitial CD4+ T lymphocyte population (IHC, anti-CD4, ×40); (**f**) isolated CD4+ T lymphocytes present in the intraglomerular area (IHC, anti-CD4, ×40). The bars indicate a size of 100 µm.

Qualitative analysis showed that the distribution of CD4+ T lymphocytes was predominantly periglomerular, with very few intraglomerular cells in the lumen of glomerular capillaries or in the mesangium. CD4+ T lymphocytes were also located in the interstitium, between the tubular structures, with a generally diffuse arrangement (Figure 1). 

#### 2.6.2. Quantitative Evaluation

The mean number of CD4+ T lymphocytes assessed in the entire study group was 111 ± 78.40/mm^2^ of renal biopsy. In the three different areas of renal biopsy, we found the predominance of interstitial (mean*_IT_* = 70.24 ± 44.87/mm^2^) versus periglomerular (mean*_PG_* = 37.69 ± 39.49/mm^2^) and intraglomerular (mean*_IG_* = 3.05 ± 3.27/mm^2^) CD4+ T lymphocytes. This distribution pattern was found for each LN class (Table 5). 

Referring strictly to the total mean values of CD4+ T lymphocytes, they are obviously higher in class VI, as these cells are approximately twice as numerous as that noted in the other LN classes. Similar results were also obtained for the mean values of CD4+ T lymphocytes quantified periglomerularly and interstitially. However, for CD4+ T lymphocytes, in all analyzed areas, no statistically significant differences between LN classes were recorded.

#### 2.6.3. Relationship between CD4+ T Lymphocytes and Semi-Quantitative Scores in Different Renal Biopsy Areas and Different Lupus Nephritis Classes 

At the level of the whole sample, the Spearman’s correlation coefficient showed no significant relationships between the RC_S and CD4+ T lymphocytes computed in all three areas (r = 0.203, *p* = 0.146), as well as separately for the intraglomerular (r = 0.221, *p* = 0.112), periglomerular (r = 0.116, *p* = 0.235), and interstitial areas (r = 0.223, *p* = 0.109). No significant relationships were identified between the TI_S and CD4+ T lymphocytes computed in all three areas (r = 0.127, *p* = 0.364) or separately for the intraglomerular (r = 0.192, *p* = 0.168), periglomerular (r = 0.064, *p* = 0.649), and interstitial areas (r = 0.163, *p* = 0.244) (Table 6).

It can be noted, anyway, that the total CD4+ T lymphocyte count was slightly better correlated with the RC_S (rho = 0.203; *p* = 0.146) than with the TI_S (rho = 0.127; *p* = 0.364) (Table 6). The weakest statistical correlation was found between the RC_S and periglomerular CD4+ T lymphocytes (rho = 0.166; *p* = 0.235) compared to the other three CD4+ lymphocyte considered categories (total, intraglomerular, and interstitial). Similarly, the TI_S had also the weakest correlation with periglomerular CD4+ T lymphocytes (rho = 0.064; *p* = 0.649) (Table 6).

### 2.7. CD8+ T Lymphocytes in Lupus Nephrites Classes

#### 2.7.1. Qualitative Evaluation

**Figure 2:**
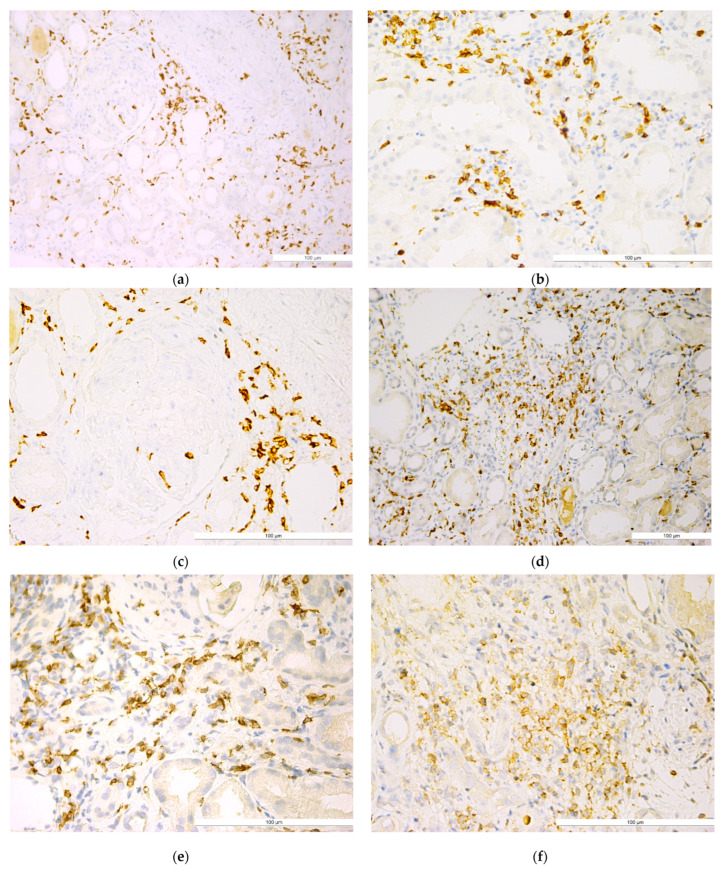
Lupus nephritis with (**a**) well-represented periglomerular and interstitial CD8+ T lymphocytes (IHC, anti-CD8, ×20); (**b)** CD8+ T lymphocytes in the renal interstitial area (IHC, anti-CD8, ×40); (**c**) CD8+ T lymphocytes with concentric periglomerular localization (IHC, anti-CD8, ×40); (**d**) CD8+ T lymphocytes in the periglomerular area (IHC, anti-CD8, ×5); (**e**) well-represented interstitial CD8+ T lymphocytes (IHC, anti-CD8, ×40); (**f**) CD8+ T lymphocytes with a tendency to form nodules (IHC, anti-CD8, ×40). The bars indicate a size of 100 µm.

Qualitative analysis showed a higher density of CD8+ T lymphocytes compared to CD4+ T lymphocytes, with a similar distribution characterized by periglomerular clustering, very few intraglomerular cells, and diffuse or nodular (primary or secondary nodules) presence in the interstitium (Figure 2).

#### 2.7.2. Quantitative Evaluation

CD8+ T lymphocytes were the best represented subset. In the overall study group, the mean number of CD8+ T lymphocytes was 578.66 ± 320.53/mm^2^ of renal biopsy. Similar to CD4+ T lymphocytes, we noted a predominance in the interstitial areas (mean*_IT_* = 372 ± 206/mm^2^), followed by a significant periglomerular number (mean*_PG_* = 195 ± 130/mm^2^), and a relatively low intraglomerular number (mean*_IG_* = 11.11 ± 16.51/mm^2^). The same distribution pattern was found for each LN class (Table 7).

Statistical analysis revealed significant differences between the LN classes for total, periglomerular, and interstitial CD8+ T lymphocytes (Table 7). Specifically, statistically significant differences were found between classes II + III versus IV and II + III versus V for total, periglomerular, and interstitial CD8+ T lymphocytes. Statistically significant differences were also noted between class IV versus V for total and periglomerular CD8+ T lymphocytes.

#### 2.7.3. Relationship between CD8+ T Lymphocytes and Semi-Quantitative Scores in Different Renal Biopsy Areas and Different Lupus Nephritis Classes 

Statistical analysis revealed a statistically significant, positive, and strong correlation between the RC_S and total (r = 0.514, *p* < 0.001) and interstitial (r = 0.557, *p* < 0.001) CD8+ T lymphocytes. The RC_S was also correlated statistically significant, positively, and moderately with periglomerular (r = 0.436, *p* = 0.001) and intraglomerular (rho = 0.341, *p* = 0.013) CD8+T lymphocytes. The TI_S was correlated statistically significantly, positively, and moderately with all four considered areas of CD8+ lymphocytes (total, intraglomerular, periglomerular, and interstitial) (Table 8). 

Specifically, for class V of LN, statistically significant, positive, and strong correlations were found between the RC_S and total (r = 0.625, *p* = 0.002) or interstitial (r = 0.717, *p* < 0.001) CD8+ T lymphocytes as well as between the TI_S and total (r = 0.512, *p* = 0.015) or interstitial (r = 0.502, *p* = 0.017) CD8+ T lymphocytes. For class V of LN, the TI_S was also statistically significant, positively, and moderately correlated with intraglomerular CD8+ T lymphocytes (rho = 0.479, *p* = 0.024) (Table 8).

### 2.8. CD4+ T Versus CD8+ T Lymphocytes in Lupus Nephrites Classes

In the overall study group, we found statistically significant differences between CD4+ and CD8+ T lymphocyte populations, as assessed by mean the number of lymphocyte/mm^2^ per each case (*p* < 0.0001). 

We also found statistically significant differences (*p* < 0.0001) between CD4+ and CD8+ T lymphocytes in all three considered areas: intraglomerular (mean*_CD4+_IG_* = 3.05 ± 3.27/mm^2^ versus mean*_CD8+_IG_* = 11.11 ± 16.51/mm^2^), periglomerular (mean*_CD4+_PG_* = 37.69 ± 39.49/mm^2^ versus mean*_CD8+_PG_* = 195.32 ± 129.92/mm^2^), and interstitial regions (mean*_CD4+_IT_* = 70.24 ± 44.87/mm^2^ versus mean*_CD8+_IT_* = 390.15 ± 271.29/mm^2^).

Statistically significant CD4+ versus CD8+ differences (*p* < 0.05) were also observed for all three locations in each LN class.

## 3. Discussion

Current research on LN aims to improve the understanding of the pathogenic mechanisms and optimize the classification system of histological lesions for higher reproducibility [34,35,36,37,38]. The involvement of the immunologic mechanism in the pathogenesis of LN is unanimously acknowledged through extensive evidence provided by experimental and human studies certifying the abnormal functionality of B and T lymphocyte populations due to their phenotypical changes, leading to aberrant immune responses [8,12,39]. However, there are only a few studies analyzing the lymphocyte morphological spectrum associated with LN-specific lesions and the potential of lymphocytes to influence disease progression [27,28,29,30,31,32,33].

In this context, our study completes our preliminary communicated results [40] focused on the analysis of the T lymphocyte profile in different areas defined in renal biopsy and in different LN classes, correlating their profile with LN-specific lesions.

Additionally, for the general characterization of the study group, we also analyzed the main clinico-biological characteristics. The statistical analysis of these characteristics showed significant differences in serum creatinine levels and eGFR, mainly due to differences between class VI and the other classes, thus sustaining the critical impairment of renal function with the onset of glomerulosclerosis. In addition, the statistically significant differences in triglycerides and serum C3 complement levels, especially between class IV and class V, could indicate changes in lipid metabolism and immunologic deficiencies, respectively, which progressively develop from the diffuse mesangial proliferation, which is specific to class IV, to the glomerular basement membrane damage, which is specific to class V.

To refine the general framework for the assessment and stratification of these lesions, we designed and applied a semi-quantitative algorithm based on two scores: the RC_S and TI_S. Using the provided score values, the algorithm ensures a more accurate assessment of the renal corpuscle and interstitial damage, and the score may be correlated with the degree of lesion intensity. The interest in defining the histologic criteria for disease staging and the assessment of activity and chronicity is justified for an accurate prognosis and optimal treatment decision. Although the ISN/RPS classification provides a set of diagnostic criteria, their reproducibility (especially in the assessment of the activity and chronicity status) is still low [36,41,42,43]. Moreover, at present, the grading of glomerular and interstitial lesions does not have the sensitivity and specificity for identifying the cases prone to end-stage renal progression and accurately predicting the disease course [37,38,44,45,46,47,48]. In this context, the revised ISN/RPS classification makes valuable recommendations for tackling such problems [36,37]. Our algorithm complements the achievements in this line of research; several parameters in our algorithm and the revised ISN/RPS classification criteria share common tasks. 

Nonetheless, it may complement the information collected using morphological activity and chronicity indexes or specific paraclinical indexes (i.e., renal SLEDAI), without diminishing their significance. Our results showed statistically significant differences among the RC_S, TI_S, and rSLEDAI score values assessed in different LN classes, thus underlining their usefulness in evaluating the morphological severity of LN. Moreover, it is worth mentioning that our results showed a statistically significant correlation between the RC_S and rSLEDAI scores assessed for the entire study group, but no correlation between the TI_S and rSLEDAI scores. These results sustain the connection between the glomerular lesions and paraclinical parameters included in the rSLEDAI score.

The analysis of the two main subtypes of T lymphocyte populations, namely CD4+ (helper) and CD8+ (cytotoxic), showed a clear predominance of the CD8+ T lymphocyte subtype, with statistically significant differences compared to the CD4+ T lymphocyte subtype. These significant differences were found both in the overall evaluation of renal biopsies and, separately, in the three considered areas, including the periglomerular, intraglomerular, and interstitial regions in all LN classes. Our results are consistent with the few studies in the mainstream research that report quantitative data on T lymphocytes within immune-inflammatory infiltrate of LN [27,28,29,30]. In addition, our findings are consistent with the immunological theory supporting the effector role of CD8+ T lymphocytes and the initiator and target role of CD4+ T lymphocytes [8,25,27]. It has also demonstrated the ability of a distinct CD4+ T lymphocyte population to stimulate the proliferation of CD8+ T lymphocytes that optimize the immune response by expressing a cytotoxic effector phenotype [8,27]. Our results support the key role of CD8+ T lymphocytes in the initiation and development of the cellular immune response, in line with other published studies confirming the increase in circulating CD8+ T lymphocytes using flow cytometry [8,30,49].

The quantification of T lymphocytes in the immune-inflammatory infiltrate can be analyzed in relation to the immune pathogenic mechanism and the prognostic value. Differences in the periglomerular, intraglomerular, and interstitial distribution of CD8+ and CD4+ T lymphocytes sustain the capacity to have a differential effect on glomerular and interstitial lesions. This observation is consistent with the hypothesis that glomerulo-nephritic lesions are the consequence of systemic autoimmune processes, whereas severe tubulo-interstitial inflammation is associated with in situ developed immunological processes [8,25,31,32].

The number of CD8+ T lymphocytes differed statistically significantly at the total, periglomerular, and interstitial levels between classes II + III and class IV and class V, separately. Additionally, for total and periglomerular areas, we found statistically significant differences between class IV and class V. These results support a progressive immune response amplification that may influence, on the one hand, the glomerular lesions and, on the other hand, the interstitial lesions belonging to class II + III as well as class V. The lack of any significant differences in their numbers in total, periglomerular, and interstitial areas between class V and class VI lesions may indicate a loss of immune response due to the extensive glomerulosclerosis and interstitial fibrosis characterizing class VI. Meanwhile, no statistically significant differences in intraglomerular CD8+ T lymphocytes were noted between all LN classes, further arguing for the distinct pathogenic mechanisms involved in glomerular and interstitial lesions. However, CD4+ T lymphocytes showed a different numerical pattern, suggesting a different behavior. In all considered areas, we found no statistically significant differences among all LN classes. This fact indicated their stability during the progression of glomerular and interstitial lesions, after CD8+ T lymphocyte activation. 

Thus, our data confirm the different roles of CD4+ T lymphocytes compared to CD8+ T lymphocytes in the LN immune response sequence [30,39,50]. It is worth noting that none of the published studies [27,28,29,30] carried out a comparative analysis of the number of T lymphocytes in three different territories and in different classes of LN. Therefore, we believe this approach supports the originality of our study, providing valuable data on T lymphocyte dynamics that may influence lesion severity in the progression of LN stages.

Nonetheless, the T lymphocyte profile was analyzed in relation to LN lesion severity, which is commonly assessed using activity and chronicity indices [36]. Data in the literature confirmed the relationship between a T4/T8 ratio of less than 1 and the activity index, but other predictive associations with any histopathological or clinical variables were missing [28]. Similarly, the CD8+ T lymphocyte number is correlated with the activity index but also with the serum creatinine level [30]. On the other hand, the total number of interstitial T and CD4+ T lymphocytes correlates with the chronicity index [29].

In our study, we used the two scores put forward by the semi-non-quantitative algorithm for assessing lesion severity as an innovative alternative to the classical activity and chronicity indexes. The statistical analysis showed that, irrespective of localization, the CD8+ T lymphocyte number correlates with the RC_S (indicating the intensity of glomerular lesions), as well as with the TI_S (reflecting the severity of interstitial changes). In contrast, no statistically significant correlations were found among the number of CD4+ T lymphocytes, the RC_S, and the TI_S in all three considered areas. 

Thus, the relationship between the two T lymphocyte populations and the RC_S and TI_S, separately, provides strong evidence supporting the major role of CD8+ lymphocytes in LN lesion progression, whereas CD4+ lymphocytes play a limited role. 

Our study has some limitations that we are aware of. First, the study’s retrospective design may introduce inherent biases in data collection. Second, although the studies focusing on LNs analyzed relatively small groups of patients (generally less than 100), our results regarding the dynamics of lymphocyte populations in relation to LN classes cannot be generalized. The direct relationship between the role of T lymphocytes in disease severity and progression certainly needs to be confirmed by more extensive studies.

## 4. Materials and Methods

### 4.1. Patients Data Collection

We conducted a retrospective study, initially including 57 consecutive patients with LN admitted to the Nephrology Department of the “Dr. C. I. Parhon” Clinical Hospital Iași between 2003 and 2008. The diagnosis of LN was confirmed by renal biopsy followed by histopathological and immunofluorescence exams in 53 cases. Four cases were excluded from the study group as the obtained tissue fragments failed to match the necessary criteria for a microscopic evaluation. 

The demographic and clinico-biological data were extracted from the patient records. This information included the following: demographic data (gender, age) and paraclinical data (urea, creatinine, estimated glomerular filtrate rate—Chronic Kidney Disease Epidemiology Collaboration (eGFR (CKD-EPI)), proteinuria 24 h, urine protein/creatinine ratio, hemoglobin, leucocytes, thrombocytes, erythrocyte sedimentation rate (ESR), cholesterol, triglyceride, double-stranded deoxyribonucleic acid (ds-DNA) antibodies, anti-phospholipid antibodies, complement C3, and the renal systemic lupus erythematosus disease activity index (rSLEDAI) score). rSLEDAI score values were calculated based on the presence of proteinuria of >0.5 g/day, hematuria and pyuria (both >5 cells/high power field), and cellular casts.

For all cases, the histopathological diagnosis was established in accordance with the LN classes defined by the revised ISN/RPS classification [36]. 

The research was approved by the Ethics and Research Committee of the “Grigore T. Popa” University of Medicine and Pharmacy Iași (15 February 2017).

### 4.2. Histopathological Exam and Lesion Assessment Using Original Semi-Quantitative Algorithms

All samples obtained by renal biopsy were assessed by two renal pathologists (I.D.C. and S.E.G) using light microscopy and immunofluorescence. The evaluation process focused on the lesions of renal corpuscles (mesangial cell and matrix proliferation, endocapillary proliferation, glomerular basement membrane changes, crescents, and partial or global glomerulosclerosis) and tubulo-interstitial components (tubular atrophy, acute and chronic interstitial inflammatory infiltrate, interstitial fibrosis, and vascular changes).

We designed and applied two semi-quantitative evaluation algorithms, the first for the renal corpuscle (RC_S) and the second for the tubulo-interstitial component (TI_S). The morphological parameters analyzed and evaluated for these scores are outlined in detail in Table 9 and Table 10. For each parameter, we obtained a severity score. For the RC_S, which is quantified as the sum of all the individual scores for each analyzed parameter, we obtained the highest value of 33. For the TI_S, which is quantified as the sum of all individual scores given for each examined parameter, we obtained the highest value of 18. The reassessment of the histological specimens was performed by two independent pathologists; differences in the individual scores given for each parameter were discussed to reach a consensus on the evaluation.

### 4.3. Immunohistochemical Exam for T Lymphocyte Assessment

The material used was paraffin blocks corresponding to renal tissue fragments obtained by renal biopsy prior to histopathological diagnosis of LN. The paraffin blocks were cut into 3 μm sections to obtain the histological specimens, which were further processed for the immunohistochemical (IHC) examination. Sections were spread on special SuperFrostPlus IHC slides and initially incubated at 37 °C for 24 h to ensure adherence to the slide. IHC staining for the identification of antigens complementary to the anti-CD4+ antibody (clone 4B12, code NCL-L-CD4-368, Novocastra, Leica Microsystem, Newcastle Upon Tyne, UK, membranous expression) and the anti-CD8+ antibody (clone 1A5, code NCL-L-CD8-295, Novocastra, Leica Microsystem, Newcastle Upon Tyne, UK, membranous expression) was performed using the BenchMark XT automated staining system (Ventana Medical System, Inc., Tucson, AZ, USA) in line with a protocol that required an initial standardization of the method. The epitope reactivation retrieval technique was used for both antibodies. The optimal dilutions for each antibody (1:40 for CD4+, 1:80 for CD8+) were determined after several runs performed to optimize the protocol. The specific membrane localization of the brown precipitate represents the positive immunoreaction. Tonsil specimens were used as external positive controls [51], and the primary antibody was omitted for negative controls.

### 4.4. T Lymphocyte Quantitative Assessment

For each case, the quantitative assessment of CD4+ and CD8+ T lymphocytes was performed in three different areas of renal biopsy: intraglomerular (CD4_*_IG_*, CD8_*_IG_*), periglomerular (CD4_*_PG_*, CD8_*_PG_*), and interstitial (CD4_*_IT_*, CD8_*_IT_*) regions. Lymphocytes within the renal corpuscle were considered for the intraglomerular area. The area adjacent to the kidney, between Bowman’s capsule and the adjacent tubules, was defined as the periglomerular area. The interstitial zone was represented by the total area of the renal biopsy, excluding the periglomerular and intraglomerular zones. The lymphocytes were counted in 5 microscopic fields selected based on the most dense cell population at 400× magnification by adapting a method reported in the mainstream literature [52]. For each case and for each area, the results were expressed as the number of positive immunoreactive cells per square millimeter. The sum of the number of CD4 and CD8 lymphocytes counted in the intraglomerular, periglomerular, and interstitial areas, separately, was considered the total number of lymphocytes.

Finally, for each LN class, we computed the mean value of CD4+ and CD8+ lymphocytes present in each of the three areas under consideration, namely, the intraglomerular, periglomerular, and interstitial areas, and the total CD4+ and CD8+ mean values are reported.

### 4.5. Statistical Analysis

The statistical analysis was carried out using SPSS 29.0 version (IBM, Armonk, NY, USA) and Microsoft Excel 2016 (Microsoft, Redmond, WA, USA). Numerical values were expressed as mean ± SD. The normal distribution of continuous values was verified by applying the Shapiro–Wilk test. For normally distributed variables, ANOVA and Student t tests were performed to assess the differences between samples. For abnormally distributed variables, the non-parametric Kruskal–Wallis test was used in the comparisons between LN classes. The correlations between quantitative variables at the level of the whole sample were evaluated using the Pearson and Spearman correlation coefficients.

## 5. Conclusions

The quantification of T lymphocytes in LN provides an opportunity to analyze their involvement in the disease progression. Our data support the different role of CD4+ and CD8+ lymphocytes in the development of glomerular and interstitial LN-specific lesions, respectively.

## Figures and Tables

**Table 1:** Baseline characteristics of patients associated with LN classes.

Clinico-Pathological Characteristics	Total (*n* = 53)
Demographic Data	
Female	44
Male	9
Age (years) (mean ± SD)	35 ± 13
Paraclinical Data (mean ± SD)	
Urea (mg%)	89.89 ± 69.66
Creatinine (mg%)	1.69 ± 1.79
eGFR (CKD-EPI) (mL/min)	68.18 ± 40.84
Proteinuria 24 h (g/24 h)	15.53 ± 46.97
Urine Protein/Creatinine Ratio	4.9 ± 4.75
Hemoglobin (g%)	10.15 ± 2.22
Leucocyte (/mm^3^ × 10^3^)	7.24 ± 4.71
Thrombocytes (/mm^3^ × 10^3^)	239.10 ± 114.67
ESR (mm/2 h)	68.27 ± 41.06
Cholesterol (mg%)	230.67 ± 79.61
Triglyceride (mg%)	199.44 ± 76.13
Anti-dsDNA (IU/mL)	209.49 ± 157.01
Anti-phospholipid (IU/mL)	16.17 ± 10.36
Complement 3 (mg/dL)	62.23 ± 26.78
Renal SLEDAI Score	11.57 ± 2.71
Lupus Nephritis Classes	
Class II	2
Class III	4
Class IV	19
Class V	22
Class VI	6

eGFR (CKD-EPI): estimated filtration rate (Chronic Kidney Disease Epidemiology Collaboration); UPCR: urine protein/creatinine ratio; ESR: erythrocyte sedimentation rate; ds-DNA: double-stranded deoxyribonucleic acid; SLEDAI: systemic lupus erythematosus disease activity index; SD: standard deviation.

**Table 2:** Relationship between clinico-biological profile and lupus nephritis classes.

Clinico-Biological Profile	Lupus Nephritis Classes				*p* Value †
	II + III m ± SD	IV m ± SD	V m ± SD	VI m ± SD	
Urea (mg%)	41.75 ± 14.59	88.5 ± 78.79	95.33 ± 72.3	110 ± 44.99	0.495
Creatinine (mg%)	1.03 ± 0.77	1.2 ± 0.78	1.54 ± 1.36	4.45 ± 3.56	0.001 **
	II + III vs. VI: *p* = 0.044 *; IV vs. VI: *p* < 0.001 **; V vs. VI: *p* = 0.004 **				
eGFR (CKD-EPI) mL/min	75.01 ± 41.45	72.98 ± 36.24	74.1 ± 43.87	24.44 ± 14.75	0.046 *
	II + III vs. VI: *p* = 0.018 *; IV vs. VI: *p* = 0.004 **; V vs. VI: *p* = 0.012 *				
Proteinuria (g/24 h)	1.7 ± 1.94	5.43 ± 5.9	30.08 ± 72.33	12.45 ± 19.6	0.458
UPCR	2.86 ± 1.27	5.55 ± 4.57	5.48 ± 5.38	1.45 ± 1.3	0.372
Hemoglobin g%	12.03 ± 1.47	10.25 ± 2.23	9.84 ± 2.09	9.6 ± 3	0.317
Leucocyte (/mm^3^ × 10^3^)	5.02 ± 2.39	8.651 ± 5.63	6.43 ± 3.74	7.62 ± 6.15	0.392
Thrombocytes (/mm^3^ × 10^3^)	247.50 ± 108.44	246.64 ± 1418.11	217.08 ± 936.67	299.20 ± 103.60	0.537
ESH (mm/2 h)	51.8 ± 29.84	58.81 ± 39.29	82.3 ± 38.71	56.5 ± 62.47	0.232
Cholesterol (mg%)	204.2 ± 48.68	239.08 ± 59.06	243.05 ± 97.55	174.5 ± 50.24	0.376
Triglyceride (mg%)	141 ± 20.45	195.54 ± 58.28	231.84 ± 85.68	131.25 ± 21.7	0.016 *
	II + III vs. IV: *p* = 0.037 *; II + III vs. V: *p* = 0.017 *; IV vs. VI: *p* = 0.016 *; V vs. VI: *p* = 0.009 **				
Anti-dsDNA (IU/mL)	118.88 ± 134.38	223.77 ± 129.52	249.98 ± 177.47	117.2 ± 123.8	0.197
Anti-phospholipid (IU/mL)	12.88 ± 10.84	22.3 ± 12.75	14.28 ± 8.26	0 ± 0	0.398
Complement 3 (mg/dL)	80.25 ± 35.56	54.27 ± 19.73	55.95 ± 21.02	91.25 ± 36.85	0.031 *
	II + III vs. IV: *p* = 0.031 *; II + III vs. V: *p* = 0.041 *; IV vs. VI: *p* = 0.004 **; V vs. VI: *p* = 0.005 **				

eGFR (CKD-EPI): estimated filtration rate (Chronic Kidney Disease Epidemiology Collaboration); UPCR: urine protein/creatinine ratio; ESR: erythrocyte sedimentation rate; ds-DNA: double-stranded deoxyribonucleic acid; m: mean; SD: standard deviation; † ANOVA test; *p* < 0.05 * statistically significant; *p* < 0.01 ** highly statistically significant.

**Table 3:** Relationship among renal corpuscle score, tubulo-interstitial score, renal SLEDAI score, and lupus nephritis classes.

Scores	Lupus Nephritis Classes				*p* Value †
	II + III m ± SD/ min ÷ max	IV m ± SD/ min ÷ max	V m ± SD/ min ÷ max	VI m ± SD/ min ÷ max	
Semi-Quantitative Scores					
RC_S	6.5 ± 2.51 3 ÷ 9	14.2 ± 3.28 8 ÷ 19	14.18 ± 6.69 5 ÷ 25	21 ± 0.63 20 ÷ 22	<0.001 **
	II + III vs. IV: *p* < 0.001 **; II + III vs. V: *p* < 0.001 **; II + III vs. VI: *p* < 0.001 **; IV vs. VI: *p* < 0.001 **; V vs. VI: *p* < 0.001 **				
TI_S	2.66 ± 2.42 0 ÷ 7	5.21 ± 2.42 2 ÷ 11	5.68 ± 3.96 0 ÷ 13	11.33 ± 1.75 9 ÷ 14	<0.001 **
	II + III vs. IV: *p* = 0.035 *; II + III vs. V: *p* = 0.045 *; II + III vs. VI: *p* < 0.001**; IV vs. VI: *p* < 0.001 **; V vs. VI: *p* < 0.001 **				
Renal SLEDAI Score	8.00 ± 3.162 4 ÷ 12	9.47 ± 2.342 5 ÷ 13	12.32 ± 2.147 8 ÷ 15	12.00 ± 2.757 8 ÷ 14	<0.001 **
	II + III vs. V: *p* < 0.001 *; II + III vs. VI: *p* = 0.021 *; IV vs. V: *p* < 0.001 **; IV vs. VI: *p* = 0.037 *;				

RC_S: renal corpuscle score; TI_S: tubulo-interstitial score; SLEDAI: systemic lupus erythematosus disease activity index; m: mean value; SD: standard deviation; min: minimal value; max: maximal value; † ANOVA test; *p* < 0.05 * statistically significant; *p* < 0.01 ** highly statistically significant.

**Table 4:** Correlation coefficients between the renal SLEDAI score and semi-quantitative scores in lupus nephritis classes.

Scores	Lupus Nephritis Classes				Total r/*p*
	II + III r/*p*	IV r/*p*	V r/*p*	VI r/*p*	
Renal SLEDAI score vs. RC_S	0.781/ 0.067	0.356/ 0.135	0.172/ 0.445	0.688/ 0.364	0.361/ 0.008 **
Renal SLEDAI score vs. TI_S	0.392/ 0.443	0.187/ 0.442	−0.088/ 0.696	0.456/ 0.364	0.239/ 0.085

RC_S: renal corpuscle score; TI_S: tubulo-interstitial score; SLEDAI: systemic lupus erythematosus disease activity index; r: Pearson correlation coefficient; *p* < 0.01 ** highly statistically significant.

**Table 5:** Distribution of the mean number of CD4+ T lymphocytes in relation to lupus nephritis class and the analyzed areas.

T CD4+ Lymphocytes	Lupus Nephritis Classes				*p* Value ‡
	II + III m ± SD/ min ÷ max	IV m ± SD/ min ÷ max	V m ± SD/ min ÷ max	VI m ± SD/ min ÷ max	
Total	93.33 ± 28.891 41.00 ÷ 121.00	110.05 ± 42.371 40.00 ÷ 192.00	90.32 ± 40.396 30.00 ÷ 167.00	207.5 ± 190.636 37.00 ÷ 496.00	0.432
Intraglomerular	1.5 ± 1.049 0.00 ÷ 3.00	3.16 ± 3.610 0.00 ÷ 12.00	3.77 ± 3.585 0.00 ÷ 12.00	1.67 ± 1.506 0.00 ÷ 4.00	0.439
Periglomerular	32.67 ± 10.520 17.00 ÷ 43.00	33.32 ± 13.115 9.00 ÷ 54.00	30.77 ± 14.700 12.00 ÷ 63.00	82.00 ± 109.148 10.00 ÷ 284.00	0.872
Interstitial	59.17 ± 23.626 24.00 ÷ 95.00	73.58 ± 35.260 12.00 ÷ 147.00	55.77 ± 25.547 17.00 ÷ 98.00	123.83 ± 92.791 27.00 ÷ 256.00	0.204

m: mean value; SD: standard deviation; min: minimal value; max: maximal value; ‡ Kruskal-Wallis test.

**Table 6:** Correlation between CD4+ T lymphocytes and semi-quantitative scores in lupus nephritis classes.

CD4+ T Lymphocytes and Semi-Quantitative Scores	Lupus Nephritis Classes				Total rho/*p*
	II + III rho/*p*	IV rho/*p*	V rho/*p*	VI rho/*p*	
T CD4+ total vs. RC_S	−0.265/ 0.612	0.310/ 0.196	0.228/ 0.308	0.338/ 0.512	0.203/ 0.146
T CD4+ IG vs. RC_S	0.136/ 0.797	0.432/ 0.065	0.251/ 0.259	0.365/ 0.477	0.221/ 0.112
T CD4+ PG vs. RC_S	0.313/ 0.545	0.168/ 0.492	0.359/ 0.101	0.338/ 0.512	0.166/ 0.235
T CD4+ IT vs. RC_S	−0.177/ 0.738	0.274/ 0.256	0.215/ 0.336	0.169/ 0.749	0.223/ 0.109
T CD4+ total vs. TI_S	0.406/ 0.425	0.140/ 0.568	0.169/ 0.452	−0.290/ 0.577	0.127/ 0.364
T CD4+ IG vs. TI_S	0.493/ 0.321	0.208/ 0.393	0.344/ 0.117	0.204/ 0.699	0.192/ 0.168
T CD4+ PG vs. TI_S	0.397/ 0.436	0.239/ 0.325	0.134/ 0.553	−0.058/ 0.913	0.064/ 0.649
T CD4+ IT vs. TI_S	0.493/ 0.321	0.064/ 0.796	0.210/ 0.347	−0.377/ 0.461	0.163/ 0.244

IG: intraglomerular; PG: periglomerular; IT: interstitial; vs.: versus; RC_S: renal corpuscle score; TI_S: tubulo-interstitial score: rho Spearman’s correlation coefficient.

**Table 7:** Distribution of the mean number of CD8+ T lymphocytes in relation to lupus nephritis class and analyzed areas.

T CD8+ Lymphocytes	Lupus Nephritis Classes				*p* Value
	II + III m ± SD/ min ÷ max	IV m ± SD/ min ÷ max	V m ± SD/ min ÷ max	VI m ± SD/ min ÷ max	
Total	146.33 ± 24.089 118.00 ÷ 175.00	481.11 ± 213.114 179.00 ÷ 870.00	794.86 ± 380.581 272.00 ÷ 1875.00	685.50 ± 358.330 81.00 ÷ 1098.00	<0.001 **†
	II + III vs. IV: *p* < 0.001 **; II + III vs. V: *p* < 0.001 **; IV vs. V: *p* = 0.013 *				
Intraglomerular	4.67 ± 7.633 0.00 ÷ 20.00	15.47 ± 23.090 0.00 ÷ 102.00	9.27 ± 12.695 0.00 ÷ 54.00	10.50 ± 6.565 1.00 ÷ 18.00	0.157 ‡
Periglomerular	51.83 ± 21.922 18.00 ÷ 76.00	142.53 ± 73.972 67.00 ÷ 276.00	262.18 ± 131.932 115.00 ÷ 632.00	260.83 ± 148.829 23.00 ÷ 428.00	<0.001 **†
	II + III vs. IV: *p* < 0.001 **; II + III vs. V: *p* < 0.001 **; IV vs. V: *p* = 0.005 **				
Interstitial	89.83 ± 7.935 79.00 ÷ 99.00	323.11 ± 151.261 78.00 ÷ 586.00	523.41 ± 322.119 129.00 ÷ 1704.00	414.17 ± 207.607 57.00 ÷ 652.00	0.002 **†
	II + III vs. IV: *p* < 0.001 **; II + III vs. V: *p* < 0.001 **				

m: mean value; SD: standard deviation; min: minimal value; max: maximal value; † ANOVA test; ‡ Kruskal-Wallis test; *p* < 0.01 ** highly statistically significant.

**Table 8:** Correlation between CD8+ T lymphocytes and the semi-quantitative scores in lupus nephritis classes.

CD8+ T Lymphocytes and Semi-Quantitative Scores	Lupus Nephritis Classes				Total r/*p*
	II + III r/*p*	IV r/*p*	V r/*p*	VI r/*p*	
T CD8+ total vs. RC_S †	−0.618/0.191	0.224/0.356	0.625/0.002 **	0.169/0.749	0.514/<0.001 **
T CD8+ IG vs. RC_S ‡	0.851/0.032 *	0.150/0.540	0.372/0.088	0.257/0.623	0.341/0.013 *
T CD8+ PG vs. RC_S †	−0.883/0.020 *	0.153/0.532	0.420/0.051	0.000/1.000	0.436/0.001 **
T CD8+ IT vs. RC_S †	−0.530/0.280	0.290/0.229	0.717/<0.001 **	0.169/0.749	0.557/<0.001 **
T CD8+ total vs. TI_S †	−0.464/0.354	0.416/0.077	0.512/0.015 *	−0.377/0.461	0.422/0.002 **
T CD8+ IG vs. TI_S ‡	0.882/0.020 *	0.131/0.594	0.479/0.024 *	−0.397/0.436	0.418/0.002 **
T CD8+ PG vs. TI_S †	−0.754/0.084	0.208/0.392	0.273/0.219	−0.485/0.329	0.332/0.015 *
T CD8+ IT vs. TI_S †	−0.522/0.288	0.401/0.089	0.502/0.017 *	−0.377/0.461	0.408/0.002 **

IG: intraglomerular; PG: periglomerular; IT: interstitial; vs.: versus; RC_S: renal corpuscle score; TI_S: tubulo-interstitial score; † Pearson’s correlation coefficient; ‡ Spearman’s nonparametric correlation coefficient. *p* < 0.05 * statistically significant; *p* < 0.01 ** highly statistically significant.

**Table 9:** Renal corpuscle score.

Quantified Parameter	Score				
	0	1	2	3	4
Damage Extent					
		Focal Lesions	Diffuse Lesions		
Mesangial Changes					
Hypercellularity	Absent	Low min 3 cells into one mesangial area	Moderate min 3 cells in 2 mesangial areas	Marked min 3 cells in >2 mesangial areas	
Matrix Proliferation	Absent	Low deposits in one mesangial area	Moderate deposits in 2 mesangial areas	Marked deposits in >2 mesangial areas	
Fibrinoid Necrosis	Absent	Absent	Absent	Present	
Sclerosis	Absent	Absent	Absent	Absent	Present
Glomerular Capillary Changes					
	Absent	2–3 inflammatory cells in 2 capillaries	Endocapillary hypercellularity	Capillary necrosis, hyaline trombi	Glomerular collapse
Basement Membrane Changes.					
	Absent	“Double contour”	“Wire loops”		Transformation into sclerosis
Segmental Sclerosis					
	Absent	Present in <50% of RCs	Present in >50% of RCs	Present in >75% of RCs	
Crescent					
	Absent	Cellular	Fibro-cellular	Fibrillar	
Global Sclerosis					
	Absent	Present in <50% of RCs	Present in >50% of RCs	Present in >75% of RCs	Present in 90–100% of RCs

**Table 10:** Tubulo-interstitial score.

Quantified Parameter	Score				
	0	1	2	3	4
Tubular Atrophy					
	Absent	Mild <25% of biopsy	Moderate >25%, <50% of biopsy	Marked >50%, <75% of biopsy	Very marked >75% of biopsy
Dilated Tubules					
	Absent	present			
Chronic Interstitial Inflammatory Infiltrate					
	Absent	Mild <25% of biopsy	Moderate >25%, <50% of biopsy	Marked >50%, <75% of biopsy	Very marked >75% of biopsy
Acute Interstitial Inflammatory Infiltrate					
	Absent	Present			
Interstitial Fibrosis					
	Absent	Mild <25% of biopsy	Moderate >25%, <50% of biopsy	Marked >50%, <75% of biopsy	Very marked >75% of biopsy
Vascular Lesions					
	Absent	Fibrinoid necrosis	Intimal fibrosis	Hyalinosis	

## Data Availability

The data used to support the findings of this research are available upon request to the authors.
